# Medical Progress

**Published:** 1870-07

**Authors:** A. N. Bell

**Affiliations:** The 47th Anniversary of the Medical Society, of the County of Kings, Brooklyn


BUFFALO

### Medical and Surgical Journal.

VOL. IX. · JULY, 1870. No. 12.

### Original Communications.

ART. I.—*Medical Progress.—An Oration, delivered by* A. N. BELL, M.D., *on the 47th Anniversary of the Medical Society, of the County of Kings, Brooklyn, April* 27, 1870.

Recognizing the importance of professional reputation as a society, no less than as individuals, we appear on this our 47th Anniversary with increased numbers and strength, to declare again our continued allegiance to a lineage as old as the civilization of mankind, and to vindicate the honor of our profession by a logical sketch of its progress.

It is the office of logic, in relation to science, to designate, systematise and justify data. Ignoring all hypotheses, logic accepts facts alone, evolves no theories, and accepts no dicta. The facts with which logic deals, however, are of two kinds, *mediate* and *immediate*—those which are derived from direct palpable cognizance of objects, and those that are demonstrable by means of and through obvious knowledge, and verifiable, for knowledge is only such in virtue of verification. Information may be gained by accident, by research, or through systematic laws, but the gaining of information is only the beginning of knowledge; the record is made by verification; and that which cannot be verified has no basis to stand upon.

Facts in medicine, as in everything else, mediately possess the essential qualities of inference by which asserted discoveries are or are not reducible to knowledge. The logical question, therefore, in regard to all mere assertions, propositions, or judgments, is not whether the assertor, proposer, or judge is worthy of credence, but, *whether in virtue of certain verifiable facts and laws*, the new assertions, propositions, or judgments are true or false.

My purpose is, for the time, to measure medicine by this standard, and, as far as practicable, without breaking the chain of my thesis, to exorcise falacies and hypotheses by the logic of facts.

Originally the Medical Art was exclusively confined to the priesthood, an hereditary caste of society among ancient nations, that embodied and monopolised all philosophy, in its broadest sense, comprehending the sum total of human knowledge.

By the researches of Oriental scholars*, in recent times, it appears that Greco-Egyptian Medicine was derived from the Hindus; that in the early ages the Hindu philosophers had numerous disciples, from Egypt and Greece, among whom they claim Plato and Pythagoras. The most ancient records of Greece are found to accord with recent researches of the East; and when the Greeks attained such a degree of enlightenment as to appreciate the arts, they sought them in the East. Medicine among the rest was transplanted, and in succeeding ages it was cultivated by a succession of men of genius, working in a field of nature under more favorable circumstances for accumulating knowledge than had previously obtained. Inquiry became the ruling spirit, and a sagacious priesthood was equal to the emergency. In Hippocrates, they stamped it with a genius by which the borrower became the owner,—the accredited originator. The oral traditions and incoherent records of diseases and remedies that hitherto had been locked up among the secrets of the priesthood, Hippocrates systematised and arranged into a text book of medicine for the schoolmen of many subsequent centuries. But in so far as the practice of medicine consists more in material things than in words, in like manner does the discoverer of remedial means deserve more honor than he who publishes the facts or reasons upon them.

Mankind had long been sensible of the importance of the knowl-

* Sir Wm Jones, M. Houdart, Thos. A. Wise, M. D. et als.

edge of remedies to assuage pain; and the discovery of means for the prevention and cure of disease was deemed to be too important a matter not to be recorded. Information of this kind was preserved, and modified or extended according to the accumulated experience of the most enlightened portion of the community—the priesthood. Hippocrates recognized the fundamental truth, that in medicine the basis of all our knowledge is the accurate observation of actual phenomena, and the correct generalization of these phenomena the sole foundation of sound reasoning. The ancient route, of which he speaks, is the route of observation and experience; and in submitting this, he made full acknowledgment of the advantage he had derived from his predecessors.

From the time of Hippocrates, there was no material progress until the establishment of the School of Alexandria, two centuries later. Aristotle had demonstrated that the heart of animals was the source of the blood vessels, but as the bans of religious reprobation obtained against the touching of the human dead body, this important fact had not been utilised. Theophrastus, a pupil of Aristotle, followed the example of his master in the study of natural history, by arranging and systematising plants.

Comparative Anatomy and Botany thus became auxiliaries of medicine, even before they had worked out their own names. The School ot Alexandria eclipsed from the first the Æsculapian temples of Greece, and Alexandria speedily became the resort of all who aspired to a knowledge of medicine. Early in the progress of the Alexandrian School, first became apparent the benefit of integration. The advanced knowledge of the natural history of animals and plants, as exclusively cultivated by Aristotle and his pupils, was, indeed, something more than a suggestive basis for the integration of medicine; it was a practical illustration of the benefit to be derived from a more enlarged field of inquiry, by which these studies were not only recognised as natural preludes to surgery and pharmacy as parts of the Medical Art, but essential to it.

The success of this Greco-Egyptian Institution, was mainly due to the collection of animals and plants, and to the authorization of dissection. But scarcely had two centuries elapsed ere Roman domination checked its progress, and by prohibiting dissection, ultimately gave it a retrograde movement. The legacy of Hippocrates became the bone of contention for innumerable sects, each claiming to be his true disciples. Men who held or taught pretended principles, acknowledged authority only unto themselves; all else was error.

Pliny, the naturalist, among the rest, derided medicine, while by his researches in natural history he added to its resources. And Dioscorides, although a dogmatic sectator, made substantial additions by a treatise on Materia Medica.

Celsus, in his work "On Medicine," evinced considerable progress, and the more remarkable, since it does not appear that he was ever a practitioner of the art, on which he was unquestionably the most accomplished of ancient authors. Indeed, Celsus' work is the only one which gives information on the state of surgery from Hippocrates to his own time; and by it we learn that many of what are now termed capital operations in surgery seem to have been understood and frequently practiced during that early period.

Previous to and at the time Celsus wrote, medicine in Rome was on a grade with the lowest occupations of a servile populace, which would seem to account for the circumstances that one so accomplished as he was, never engaged in its practice. His knowledge was wholly of foreign origin, notwithstanding he wrote at Rome, and he seems to have regarded the wrangling sects of his native countrymen who called themselves physicians, as beneath his notice. The riot of ignorance continued to rage for a little more than a century later, when it was attacked by the immortal Galen. Unlike Hippocrates in being surrounded by an appreciative fraternity of learned men, he stood alone as it were, in the midst of pretenders and revilers, whose every effort tended to debase the art which he loved. Possessing superior advantages of birth and education, he used them with energy. He laid out and accomplished for himself a broader scope and higher degree of scholarship in all the learning of the period, than any one that had preceded him. He studied philosophy and the arts in the schools of highest repute, while he scrupulously kept aloof from contending factions. His knowledge of medicine he principally acquired at Alexandria, after which he traveled through various countries for the express purpose of gaining information. Finally secure in his acquirements and confident of his power, he devoted himself scarcely less to the exposition of imposters than to his profession. And so far was he in advance of his contemporaries, that the futility of their reasoning served but to display his superiority. The result was, that on all subjects connected with medicine, he acquired a hold on the public mind that has never been equalled by any other physician. Praxagoras had previously distinguished the arteries from the veins, but the arteries he considered air vessels. Aristotle and Erisistratus adopted and taught this view. Galen refuted it, by showing that the arteries being wounded always gushed blood. And he turned this knowledge to good effect, by tying them for the purpose of stopping hemorrhage. He also showed progress by his accurate knowledge of the human skeleton, and the organs of motion. He demonstrated the muscular filaments, and the ramification of the blood vessels and nerves between them. He traced the nerves of the brain and spinal marrow, and, by experiments on animals, he distinguished the nerves of sensation and motion. He enumerated and classified the nerves into pairs and groups, and discovered several of the ganglia. He recognised the natural divisions of the human body into cavities, and was familiar with the locality and appearances of the chief organs; indeed, his knowledge of particular structures was general, and, in the main, so correct as to constitute the basis of all subsequent anatomical classification. Galen's chief means of acquiring this knowledge, appears to have been the vivisection and dissection of animals; for, while he is authority for human dissection having previously been practised by others, we have no evidences dences that he ever practised it himself. But we have evidence that in the latter part of the second century of the Christian era, shortly before the downfall of the Alexandrian School, and about the time of Galen's attendance there, Herophilis and Erisistratus not only dissected the human body, but that they also vivisected condemned criminals.* Galen also contributed to improve therapeutics, by pointing out the two-fold (primary and consecutive) effects of many medicines; and diagnosis, by defining the shades of difference in the pulse. And to Galen's works, especially, are we indebted for the preservation of the Medical Art for many subsequent centuries. In the final complete destruction of Alexandria, by the

* History of Medicine, from its origin to the 19th century, translated from the French by C. G. Comegys, M.D., Professor, etc., Philadelphia, 1867. Renouard, p. 179.

Arabians, in the seventh century, "some books escaped from the general wreck of literature and science; and there were not wanting some individuals capable of estimating their value. Among these relics were the writings of Galen; and, at an early period of the Saracenic Empire, these books began to be held in very high estimation. They were translated into Arabic; were commented upon in various ways, and soon acquired a degree of celebrity, scarcely short of what they had previously enjoyed among the Greeks themselves.*"

The first reproduction of Galen's writings, by the Arabians, was by Rhazes, about the end of the 9th century. The only progress indicated by him, is the free employment of what were called chemical remedies, and his description of small pox and measles. For his knowledge of small pox, however, he refers to an Alexandrian physician, named Ahrun, who had described it nearly three centuries before.

Avicenna, two centuries later, gained a reputation, by his medical writings, for great profundity of learning, while his highest ambition seems to have been a thorough knowledge of the works of Galen, which he believed to contain the sum of medical wisdom. He made a large collection of material from all quarters, almost regardless of its value. And it is particularly notable that, in his chapter on leeches, he acknowledges Indian authority; and his description is word for word that of Susruta, who wrote a thousand years before the Christian era.† Indeed, all of the accredited improvements made by the Arabians seem to have been derived from the same source. They were gatherers and conservators, but not originators. And by the intervention of the crusades between the Asiatics and Europeans, the Arabians only gave back in a new dress, that which they had derived, in the first place, from the relics of Alexandria, and that which they obtained from India.

The first Christian prince who concerned himself with medicine, was Roger, the founder of the kingdom of Sicily, A.D., 1140. He proclaimed an ordinance, requiring every person who purposed to devote himself to medicine, to obtain authority from a magistrate,

* Cyclopœdia of Practical Medicine, vol. 3, p. 200.

† Boyle, Asiatic Journal, vols. 3, 4 and 5.

† Review of the History of Medicine, by Thos. A Wise, M.D., 2 vols., London, 1867.

under pain of confiscation and imprisonment. From this time many other princes followed his example, in establishing regulations and restraints, which, in the end, led to medical organizations, and the institution of faculties and universities. In the thirteenth century, Frederick II., grandson of Roger, issued an edict, in virtue of which no one could practice medicine in the kingdom of Naples who had not been examined and created a master in the School of Salerno. To effect this, candidates were required to be of legitimate birth, to be twenty-five years of age, to have studied logic three years, medicine,—including surgery—five years, and to undergo public examination on the therapeutics of Galen and the aphorisms of Hippocrates; which, being satisfactory, an oath was administered for faithful conduct,—to submit to the rules regulating practice, not to share the profits of the apothecary, and to gratuitously attend the poor.

The School of Salerno flourished from the eighth to the thirteenth centuries, and is said to have been founded about the middle of the seventh century, by some of the professors who escaped from Alexandria when it was overrun by the Saracens.

In the time of Charlemaigne, the cathedrals of France, generally, possessed schools, where writing, reading, arithmatic, singing, theology, and sometimes, medicine, were taught; and before the portals medical advice was given to the poor. From mere medical advice, in the first place, sprang the practice of dressing wounds, and simple surgical operations. On account of this, about the middle of the twelfth century, the Pope declared the practice of surgery incompatible with the priestly office, and forbad every bloody operation, on pain of excommunication; but in order to still maintain jurisdiction over learning, medicine was made a branch of university education, and it was thus that during the thirteenth century most of the great universities of Europe were created. Practical medicine still remained in the hands of the priests, the only men who, at that time, could maintain pretention to learning, while surgery was abandoned to the ignorant and irreligious barbers, bathers and bonesetters. who were looked upon with such contempt as to be excluded by birth from apprenticeship to an artisan.

Early in the fourteenth century, the School of Salerno was excelled by that of Bologna, by the revival of the dissection of the human body. In A.D. 1315, Mondini, a professor in the School of

Bologna, dissected the bodies of two women, and shortly after wrote an account of it, illustrated with drawings from nature. To him, therefore, belongs the credit of being the first to add pictorial illustration to anatomical description. His dissection, however, was scarcely less crude than that practiced by the ancient Hindus. "Beneath the veins of the forearm," he remarks, "we see many muscles, and many large and strong cords, of which it is not necessary to attend in the anatomy of such a corpse (a recent one), but on one dried in the sun for three years, as I have shown otherwise in developing the number and the anatomy of those of the superior and inferior extremities. He takes an opposite and unnatural course to discover the nerves, advising maceration in running water.* " For more than a century later, no one dared to follow the example of Mondini, in applying the dissecting knife to the human body. Mondini's conscience seems to have smote him for his first attempt, since he did not venture to open the skull, for fear of committing mortal sin. And his scruples were more than confirmed by the bull of Pope Boniface, forbidding eviseration and anatomical cooking, a practice which Halfinx states was only intended to prohibit the custom of the Crusaders of cutting up and boiling the bodies of their relatives deceased in infidel countries, so as to send them to their burial places in holy ground! It is certain, however, that the same bull was interpreted as prohibiting the dissection of the human body, for, in 1482, the University of Tubingen had recourse to the authority of Pope Sixtus IV., to obtain permission for dissection.†

During the middle ages, charitable institutions, hospitals and medical schools were extensively multiplied; and popes, princes and priests gave examples of devotion to the religious sentiment of the age, by personally attending the diseased and wounded. During the general prevalence of plague and other epidemics, public baths were instituted in many cities, and this measure alone, probably, did more to appease the ravages of plague than all other means combined. War, famine and disease concurred to test the zeal of devotees, to a degree unequalled at any other time. "During the period of universal distress, the monasteries of Constantinople had

---

* Renouard.—First practised by the Hindus.—WISE.

† Hist. de l'Anatomie, lib. v., part IV.—Renouard, p. 294.

been, for some time, the refuge of the learned men who had been driven from Italy, by the perpetual wars in which that country had been engaged. They had taken with them what they had considered their most precious treasures,—the manuscripts of the ancient classical writers—probably regarding them more as objects of curiosity than of real importance. These manuscripts had now been buried for a long time in their libraries, their existence being unknown to the rest of the world. When the Monks were expelled from their retreats by the Turks, and flying into Italy, they carried back with them their manuscripts. A spirit of improvement had already begun to manifest itself in Italy, which was considerably incited by their guests, who, in their turn, by their change of situation, and by the new society into which they were introduced, became more aware of the value of their literary treasures; while their own acquirements, limited as they were, gave them a degree of respect with their new associates, which tended to inspire them with a desire of further improvement.* " It was thus that, A.D. 1483, Mahomet II., having undertaken to destroy all the treasures of Greek learning, was instrumental in preserving them. The art of printing had been invented but a few years before, and the Western world was thus supplied with an amount of the most valuable material. The taste for Greek literature, which had already begun in Italy, was cultivated with such ardor that the Arabic was speedily displaced. The monuments of Greek and Latin learning were collected and published, and the pathway of science paved with imperishable lore. In addition to this, the art of printing supplied the means of circulating monographs of particular diseases and cases, with the reports of hospitals and other institutions for the care of the sick. These early publications on medical subjects, although intrinsically of little worth, served to reestablish a habit of observation, and to direct attention to facts as the foundation of true medical philosophy.

At the beginning of the fourteenth century, Pitard founded a College of Surgeons, in Paris, out of the Brotherhood of St. Cone, a society of barbers, which occupied a sort of middle ground, above the barber surgeons, and below the physicians, and in communion

* Ackermann, ch. xxxii. Cabanis,—Cyclopœdia of Practical Medicine, vol. iii., p. 206.

with both. In the course of a century, this institution had so prospered, that it was admitted to a union with the university, and from that moment, says Malgaigne, a new state of things commenced. The medical schools of Padua, Pavia, Milan, Rome, Naples and other cities, all acquired celebrity; yet they confined their curriculum almost exclusively to the works of Galen. In the latter part of the fifteenth century, Thomas Linacre, of Canterbury, who is said to have been the first of his nation who learned to speak purely the language of the Romans, perfected his studies in Italy, and was, soon after, made physician in ordinary to Henry VII. and Princess Mary. Through his learning and translations of the works of Hippocrates and Galen, two chairs were founded at Oxford and Cambridge, the incumbents of which had to explain the writings of these ancient worthies. Linacre was also the chief instrument in founding the College of London. At that time, the bishops alone had the right of bestowment of medicine in their dioceses; whence it was that the practice of medicine continued in the hands of monks and illiterate empirics. Linacre had need of all his learning and position at court, to do away with these prerogatives of the bishops; but his perseverance and enlightened zeal were equal to the task. He obtained the issue of letters patent, prohibiting the practise of medicine by any one who had not received his degree in one of the two universities in the kingdom, and who had not been examined by the president of the college at London, assisted by three physicians, delegated expressly for that purpose. Chiefly through the efforts of Linacre, celibacy ceased to be obligatory on medical men, and ecclesiastical benefices were no longer sought as desirable for a physician.*

In 1536, Ambrose Parè, a barber surgeon, of Paris, made his first campaign as surgeon to Marshall de Monte. Freed from the yoke of authority, which had for centuries environed medicine with superstitious blindness, he determined to observe for himself, and to accept the suggestions of experience, based upon accurate observation, as paramount to the doctrines of the ancients. Galen's method of tying the vessels for the purpose of stopping hemorrhage, which had long since fallen into disuse, Parè restored; and he was,

---

* Friend.

probably, the first to employ the ligature after amputations, instead of the actual cautery.

Hitherto, gunshot wounds were thought to be poisonous, and were treated by the application of boiling oil, hot pitch, or red hot iron. Among Parè's first observations, was, that wounds which had escaped these severe applicatiens were the better for it, and from that time he discarded them. Notwithstanding, barber surgery continued to prevail in its most contemptible aspects.

In 1542, the barber surgeons of London were, by act of parliament, raised to a dignity similar to that of the College of St. Cone, of Paris, under the name of "Company of Barbers and Surgeons." The same act directed that the masters, or governors, of the said company, should have at their free liberty and pleasure "the bodies of four felons to experiment upon.*" It is uncertain whether dissection was forthwith proceeded with in England; but, in 1566, anatomical demonstrations were made at stated periods, in a public hall set apart for that purpose; and there was also a readership of anatomy held by physicians appointed by Royal authority.

Early in the sixteenth century, the popes, who still stood at the head of scientific movements, withdrew their interdictions of anatomy, and the universities of Italy gave the first examples of dissection. Achilgini, Benedetti, and perhaps also, Jacqes Berrenger, dissected at Bologna and Padua, previous to A.D. 1500. But there was no substantial progress until about the middle of the sixteenth century. Vesalius was the first after Parè, who dared to subordinate Galen, and show his errors, on the ground that the greater part of Galen's dissections having been of monkeys and other animals, did not represent the human structure. This made him many enemies, but also some friends and followers. Dissection, which had at the first been pursued privately, was now practiced openly, in amphitheatres erected for the purpose. Anatomical chairs were created, and paid out of the public treasury, and permission given to use the dead bodies of criminals not only, but others. Human intelligence, long buried in a lethargic sleep, gradually roused up, and signified its rising by discoveries of the highest importance. Learning emerged from the cloisters, and once

* Chelius Surgery, vol. I., p. 19, Philadelphia, 1847.

more showed itself abroad as in the days of Pythagoras, but with means of improvement far more numerous and efficient. Greek and Latin literature was exhumed from the dust of the convents, and studied with avidity. Soon these monuments of ancient learning were found to be insufficient to satisfy inquiry. Criticism, more and more severe, revealed numerous defects, and finally stripped these still venerated relics of their sacred character, and assigned them a place from which they might henceforth be improved upon, and perfected in proportion to the advancement of human intelligence. The scalpel took the place of the razor, and the foundation of rational medicine was laid in anatomy. Plates made muscular fibre distinguishable from nerve expansion; lymphatic vessels appreciable; the muscular structures of the heart apparent; and the circulation of the blood became a desideratum. The pulmonary circulation was almost discovered in 1550, by Servetus. He denied that any blood passed through the septum, as then taught by others, and asserted that it all passed through the lungs, by the pulmonary artery, and returned by the pulmonary veins. Andrew Cesalpine had discovered and taught, that "the openings in the heart are disposed in such a manner, that the passage is free from the vena cava into the right ventricle, and from that cavity into the lung; a communication from the lung to the left ventricle, and from this last, into the aorta. Membranes are placed at the orifices of the various conduits in such a way, that a retrograde flux of the liquid column is impossible.*" Aided by Aquapendente, he demonstrated the course of the blood through the lungs, and added that the last or most minute ramifications communicated with the veins; that the blood passed from the arteries into the veins during sleep, which he inferred from the swelling of the veins, and the diminution of the pulse at that time. The valves of the veins were also known; and it had been ascertained that the ligation of an artery stopped the pulse below, and that a ligated vein shrunk above the ligature. Among the most diligent students of anatomy, of the time, was William Harvey. Returning to London, from Padua, where he had studied the requisite five years for his degree, he entered upon professional life, while he continued to prosecute the study of anatomy, making, in conjunction with his dissections, a large number of

* Renouard, p. 297.

vivisections. Possessed of all the facts that had previously been made known, and endowed with a spirit of inquiry and perseverance, equal to the importance of his pursuit, every step of his progress was a crucial experiment. Thoroughly embued with the idea that certain truth could only be known by the regular and constant succession of one phenomenon to another, he was content to labor many years for its accomplishment. Gradually the object he sought revealed itself to him, and took form. Fortified alike with a knowledge of the falacies, as well as the facts of all who had preceded him in the same field of research, he hesitated not to declare the whole truth, and to defend it against all opposition. The whole process of Harvey's researches was in accordance with the principle of "induction upon data carefully collected and considered." The motion of the blood and its existence in the arteries and veins, had been long known; but the important fact that it made a continuous transit from the arteries into the veins without interruption, was reserved for Harvey. Yet he never *saw* this. He knew not *how* the blood got from the arteries into the veins. But the fact he was sure of, by induction, as a necessary consequence of mediate data he had carefully collected and considered. Harvey lived to see his discovery universally accepted, though it was not fully demonstrated until three years after his death, by Malpighi, who, in 1661, demonstrated, for the first time, by aid of the microscope, the progression of the blood through the capillaries.

Until about the time of Harvey, it had been the practice for many centuries, to place at the head of all philosophical treatises certain axioms improperly named "principles," as an index of the writer's purpose. And the axiom of Aristotle, "*Nihil est in intellectu quod non prius fuerit in sensu,*" had been received as the quintessence of scholastic philosophy. But at the beginning of the seventeenth century, scientific progress had so far comprehended scholastic philosophy as to shake to the surface all that was of value; and we find medical philosophers practicing the reverse of Aristotle's dogma. To seize upon, and combine this new process of improvement into a system, was the work of Francis Bacon; but, that "particular ideas are the basis of the pyramid, and axioms the summit; to study and apply what is known to the discovery of new truths by induction, by which our senses are guarded against error;"* that these ideas were actuating principles long before they were enunciated by the great philosopher, admits of no question. And it has been truly remarked, that had Bacon been wanting an example to illustrate the truth of his doctrine, it would not be easy to adduce a more striking example of the way in which ultimate rational truth is arrived at, by a succession of inferences, than is contained in Harvey's Essay on the Heart and Blood.†

The spirit of Harvey was contagious. The process by which he had arrived at such an important truth opened up new fields of research. The discovery of the circulation of the blood was, after dissection, in a great measure due to the vivisection of animals, the only means, other than experiments on the human body, by which such knowledge is attainable. There was, at that time, a sentimental criticism of Harvey's experiments, such as has frequently manifested itself from various quarters since. To intelligent minds, generally, the importance of Harvey's discovery and its congeries, are an all-sufficient reason. But, unfortunately, for the good of scientific inquiry, there always exists a respectable number of persons who, incapable of appreciating the motives of profound inquiries for the good of mankind, arrogate to themselves the sum of humanity. They cannot be made to see that every step taken in the science of man's life, is an important part of the progress that adds to the sum of human happiness for both the present and the future.

Shortly after the time of Harvey's discovery, Asellius, of Milan, while examining the abdominal viscera of a dog that had been killed soon after eating, discovered the process of nutrition,—the *lacteal* circulation. His discovery, like Harvey's, had been preceded by certain intimations, as it were, by Fallopius and Eustachius, half a century before; but neither of them comprehended anything of the use, or the relation of the lacteal vessels to the sanguiferous system. Asellius regarded the lacteals as having a distinct function, and traced them to their origin in the mucous surface of the intestines; but their termination in the thoracie duct, was not discovered until thirty years later, by Pocquet. The lymphatic and absorbent systems were discovered in a similar manner, by other observers.

* Nov. Organ: lib. 1, ch. 1.

† Works of Harvey, by Robert Willis, M.D. Introduction.

These discoveries led to the use of high magnifying powers in anatomical researches, the pioneers being Malpighi and Leeuenhoec. By this means they were the first to demonstrate the movements of the blood discs in the capillaries. By the same means, De Graaf, Mayo, and others, made important progress in the knowledge of respiration, generation and gestation; and Vieusens, Willis, Pinel, Kepler, and others, improved our knowledge of the nervous system. Meanwhile, there were "Mechanical," "Chemical," "Metaphysical," and other sects, and numerous individuals, as at the present day, who professed to possess certain cures for all the diseases that flesh is heir to, without any rational knowledge of the human organism, or of the remedies which they used. Joubert, a sprightly writer of the time, after having shown the presumption of so many persons assuming to act as physicians, without the requisite knowledge, narrates the following adventure:—"It is said that the duke of Ferrara, Alphonso d'Este, at one time proposed, in a familiar way, the question,—'In what calling are most men engaged?' One said, 'Shoemakers;' another, 'tailors;' a third, 'carpenters, masons, pettifoggers and laborers.' Gonelle, his famous buffoon, said there were more 'physicians' than any other class of men, and made a bet with the duke, who denied it, that he would prove it in twenty-four hours. The next morning, Gonelle left his lodging, wearing a great night cap, and a cravat tied round his chin; then a hat over all, and his mantle drawn up over his shoulders. In this attire, he took a route through a populous street leading to the palace of his master. The first person he met asked him what was the matter? He replied that he had a raging toothache. "Ha, my friend," said the stranger, "I know the best remedy in the world against it," and told it to him. Gonelle inscribed his name on his tablet, pretending that he was writing down his recipe. A step further on, he found two or three together, who all asked the same question, and each one gave him a remedy. He inscribed their names, as the first. And thus he pursued his course, very gingerly, to the end of the street, not meeting a single person who did not offer him a recipe, and different from the rest, each one assuring him that his remedy was established, certain and nearly infallible. He wrote down the names of all. Approaching the lower court of the palace, he found himself surrounded with gentlemen (for they all knew him), who, after having learned his affliction, compelled him to take their recipes also, which each one assured him was the best in the world. He thanked them all, and wrote down their names. When he entered the chamber of the duke, his excellency cried out, "Eh! what is the matter, Gonelle?" He replied, very piteously and complainingly, "The toothache, the worst that ever was." To which his excellency replied,—"Ha! Gonelle, I know a thing which will drive off the pain at once, without touching the tooth. Mr. Antonio Musa Brussavolo has never employed a better one. Do so and so, and immediately you will be healed." Gonelle suddenly threw off his head dress, and his other attire, crying out, "And you, also, my lord, are a physician!" Look at my list, and see how many others I have found, between my lodging and your palace. Here are nearly two hundred, and I have not passed through one street. I will engage to find ten thousand in the city, if I go everywhere. Find me as many persons in any other business." *

(*To be continued in our next.*)

* Erreurs populaires par Laurent Joubert, 1587.

